# Supplementary material for: Radio frequency interface quality assessment in 4G/5G: Effects of IQ imbalance, phase noise, and nonlinearities on error vector magnitude
Source: PLoS One. 2025 May 22;20(5):e0324170. doi: 10.1371/journal.pone.0324170 (PMC12097624; doi:10.1371/journal.pone.0324170)
Supplement: Attachment — Submitted filename: renamed_e3db8.docx [file pone.0324170.s002.docx]

**Responses to Reviewers’ Comments**

**Reviewer 1**

Dear Reviewer, we sincerely appreciate your qualified and well-reasoned review of our article. We highly value your insightful comments and recommendations. Please allow us to provide the following responses to your remarks and suggestions.

1) On the one hand, the manuscript is generally well-written, but some sentences could be restructured for better readability. A minor language revision would improve clarity. It is especially important for authors to check all spelling hyphens that appear in the text. Many of them are unnecessary, or are simply incorrect.

*Responses to comments:*

We thank the reviewer for their valuable feedback regarding the language quality of the manuscript. The text has been carefully proofread by the authors, including with the support of automated grammar and style tools such as Grammarly. We will also make sure to work closely with the journal’s editorial team at the production stage to ensure that the language meets the highest academic standards. We appreciate your attention to this aspect and will remain attentive to every detail. Regarding the hyphens that appeared in the text. Thank you, we have corrected them.

2) On the other hand, some figures, such as those showing spectral characteristics, could benefit from higher resolution and clearer labels for better visualization.

*Responses to comments:*

Dear Reviewer, we sincerely appreciate your keen attention to detail and insightful observations regarding the visual presentation of our graphical materials. Regarding the quality of illustrations, all graphical materials in the manuscript are direct exports from the MATLAB environment. They clearly depict dependencies, axis labels, and other attributes, meeting the requirements for visual clarity. We acknowledge the importance of high-quality visuals and have made every effort to enhance the resolution and readability of the figures, particularly those illustrating spectral characteristics. Additionally, we have carefully reviewed all illustrations (Fig. 2, Fig.6, Fig.9, Fig.10) to ensure optimal clarity and precision. If necessary, we remain open to making further refinements to meet the highest standards of visualization.

3) Finally, it would be remarkable if the authors consider validating the Simulink results with empirical hardware measurements.

*Responses to comments:*

Dear Reviewer, thank you for taking the time to review our manuscript and for your constructive comments. Please allow us to address them as follows. We acknowledge that our research is based exclusively on simulation results. This approach was deliberately chosen as it allows for studying the impact of radiofrequency distortions under controlled conditions, which is particularly critical during the initial stages of analyzing complex systems. The simulation results not only provide a foundation for further studies but also create a roadmap for identifying key performance metrics for future experiments. Indeed, high-level research often relies on results obtained through experimental setups. However, the extensive and complex studies conducted within our work demonstrate the importance of the simulation-based approach. It enables us to pinpoint intricate and challenging aspects that require focused attention during subsequent experimental investigations. This approach ensures:

1.	Preliminary analysis of the efficiency of novel solutions, such as 256QAM, massive MIMO, AI, adaptive PLL circuits, etc.

2.	Resource optimization by focusing on critical aspects before initiating physical experiments.

3.	Establishing a basis for experimental validation of the obtained results.

In future studies, we plan to perform experimental testing using hardware prototypes to validate the findings presented in this work. We fully agree that conducting experimental research in real-world settings is a crucial step in validating the practical relevance of our results. We plan to proceed with field experiments after completing the modeling, analysis, and evaluation of results based on existing Simulink models and the hardware setup, including the QM78207 chipset. The findings presented in this paper will serve as the foundation for conducting real-world experiments and providing a platform for analyzing the impact of RF impairments on various parameters, particularly on Error Vector Magnitude (EVM). We also plan to conduct field experiments in diverse environments (urban, rural, and indoor settings) to assess the practical impact of RF impairments on system performance. This will provide us with a more accurate understanding of the real-world conditions and add significant value to the results of our research. This ensures that the content is valuable not only for a narrow audience of experts familiar with the subject but also for a broader range of specialists, including those in related fields. Such an approach promotes better understanding and facilitates interdisciplinary knowledge dissemination.

4) The discussion section effectively connects the results to real-world applications, but it would be helpful to explicitly mention any limitations of the study and potential areas for future research.

*Responses to comments:*

Dear Reviewer, thank you for your thoughtful and well-reasoned comment, as well as your suggestion to highlight certain limitations of the presented study and outline potential areas for future research. In response to your remark, we have further elaborated in the "Discussion" section on aspects related to the study's limitations and have identified promising directions for further investigation.

**Added to the Discussion section:**

In this context, we would like to highlight certain limitations of the presented study and outline potential areas for our future research. Despite the valuable insights obtained, this study has several limitations that should be addressed in future research. First, the analysis was conducted under controlled laboratory conditions, which may not fully capture the complex effects of real-world interference, varying environmental factors, and hardware imperfections encountered in practical deployments. Future studies should incorporate field measurements and dynamic channel conditions to validate the findings in real-world 5G network scenarios, thereby improving the applicability of these results in operational environments.

Second, while the study focuses on key hardware impairments such as IQ imbalance, amplifier nonlinearity, and phase noise, other factors, including mutual coupling in massive MIMO arrays and power amplifier memory effects, may further impact signal quality. Investigating these additional impairments would provide a more comprehensive understanding of RF system behavior, and the interplay between various hardware distortions in real-world systems remains an area requiring further exploration.

Moreover, this study primarily evaluates Error Vector Magnitude (EVM) as a performance metric. While EVM is a critical indicator of signal quality, it does not account for higher-layer performance metrics, such as Bit Error Rate (BER) and Frame Error Rate (FER), which are essential for assessing end-to-end system performance. Future research should investigate the correlation between EVM and these metrics to better understand the practical implications of RF impairments on overall network reliability and user experience.

In addition, this study highlights the potential of AI-driven adaptive algorithms for real-time compensation of RF impairments, but further investigation is needed to develop and validate machine learning-based models capable of dynamically adjusting RF parameters in response to changing network conditions. The practical integration of such solutions into 5G and beyond-5G networks remains an open challenge, with many technical and operational hurdles to overcome.

Lastly, as the field of communication technologies advances toward 6G, emerging technologies such as ultra-massive MIMO, terahertz communications, and reconfigurable intelligent surfaces (RIS) will introduce new challenges related to RF impairments. Future research should explore how the findings of this study can be extended to next-generation systems to ensure the continued enhancement of signal integrity and spectral efficiency in the face of these novel technologies.

**Reviewer 2**

Dear Reviewer, we sincerely appreciate your qualified and well-reasoned review of our article. We highly value your insightful comments and recommendations. Please allow us to provide the following responses to your remarks and suggestions.

1) The novelty of the proposed work is difficult to understand. Most of the conclusions are from the earlier reported work.

*Responses to comments:*

Dear Reviewer, we sincerely appreciate your insightful and meticulous review of our work. Your keen observation regarding the novelty of our study has prompted us to further clarify the unique contributions of our research.

Following an extensive analysis of the works of leading authors published in top-tier journals, we identified significant gaps in the existing body of research. Most prior studies have not provided a comprehensive evaluation of the impact of multiple RF distortions on EVM in 4G/5G systems. Specifically, earlier works tend to focus on isolated distortion effects—such as IQ imbalance or amplifier nonlinearities—without addressing their combined impact alongside phase noise, channel noise, and signal coding characteristics. Furthermore, many previous studies lack a direct connection between distortion analysis and practical performance metrics, such as throughput, packet loss, and real-world RF quality parameters.

Our research advances the field by bridging these gaps through a holistic approach to distortion modeling and quantitative assessment. Unlike previous studies, we:

1) conduct a comprehensive Simulink-based simulation that integrates multiple RF distortions, including IQ imbalance, phase noise, power amplifier nonlinearities, and channel noise, in a unified framework.

2) quantitatively evaluate the impact of these distortions on EVM, providing a more detailed and practical analysis than existing theoretical models.

3) integrate a hardware-based perspective by considering the QM78207 chipset, ensuring that our findings align with the latest industry developments and modern RF interface design challenges.

4) offer insights that directly contribute to optimizing RF front-end designs for improved communication quality in 4G/5G networks.

By addressing these overlooked aspects, our study provides not only a deeper understanding of RF distortions but also practical methodologies for mitigating their effects in real-world telecommunication systems. We believe that this contribution significantly enhances the relevance and applicability of our work in optimizing RF performance and supporting the evolution of next-generation communication technologies.

We appreciate your valuable feedback and hope this clarification effectively addresses your concerns regarding the novelty of our study.

2) Though hardware demonstration could add significant appreciation, the simulation framework need to be highlighted with major contribution from the authors.

*Responses to comments:*

Dear Reviewer, thank you for your valuable comments. We fully understand the importance of hardware demonstration for additional verification of the results. However, we would like to emphasize that the primary contribution of this work lies in the enhancement of the simulation methodology, particularly in the development of a comprehensive RF receiver and transmitter model within the Simulink environment. Our study provides key insights into the impact of factors such as I/Q imbalance, phase noise, amplifier nonlinearities, and their interactions on signal transmission characteristics and system performance.

While hardware demonstration could indeed significantly enhance the appreciation of the work, we emphasize that the proposed models allow for a detailed analysis and evaluation of the effects of various impairments without the need for expensive and complex physical experiments. Modeling such complex effects within Simulink significantly simplifies the process of developing and optimizing system parameters for LTE and 5G without requiring physical prototypes.

Our study demonstrates how comprehensive simulation can provide an accurate representation of real-world operating conditions, especially for key parameters such as EVM (Error Vector Magnitude) and SNR (Signal to Noise Ratio). An important aspect of our work is also the modeling of the mutual influence of all these factors on signal quality and system efficiency, which is crucial for high-speed communications such as 5G. This approach provides valuable insights for further improvements under real-world conditions without the need for intricate physical experiments.

Additionally, during the study, we investigated the impact of I/Q imbalance, phase noise, and amplifier nonlinearities on the performance of LTE and 5G systems. This allows us to identify key points for the further optimization of transmitter and receiver characteristics in real operational environments.

Thus, although hardware demonstration could be beneficial for verifying the results, we believe that the proposed simulation methodology in Simulink makes a significant contribution to the development of advanced approaches for signal quality assessment in modern mobile communications. It helps identify potential issues and areas for improvement, which can later be realized in real systems.

We believe that our work provides useful and practical recommendations for the development and testing of equipment for future mobile communications, and represents an important step toward the enhancement of LTE and 5G systems.

Once again, we thank you for your comments and observations.

3) The manuscript quotes chapter 3 at two different locations for which the reference should be available.

*Responses to comments:*

We sincerely thank the reviewer for this observation. The references to "chapter 3" in the manuscript are internal and refer to the subsequent section of our own article, titled “3. Results”, rather than to an external source. These mentions were intended to guide the reader to the detailed presentation of the experimental findings that follow. To avoid any misunderstanding, we are ready to clarify this wording if necessary (e.g., by replacing "chapter" with "section") to ensure better readability and precision.

**A new version of the sentence:** This will be demonstrated in section 3 of this article, where we present the results of experimental studies.

**A new version of the sentence:** The research results presented in section 3 of this article below highlight the importance of continuously improving the methods for assessing the quality of RF interfaces to ensure the reliability and efficiency of next-generation communication systems.

4) Please explain fig. 4 and fig. 5 clearly with all the clarifications of the arrows and symbols presented.

*Responses to comments:*

Dear Reviewer, we sincerely thank you for your valuable comment regarding Figures 4 and 5, particularly your suggestion to provide a more detailed explanation of the purpose of each block within the Simulink-based model.

Below, we present a clarification of the block functionalities, along with explanations concerning the specific features of working with the proposed model components, including the use of arrows and icons to access internal structures and parameter configurations.

**We have added clarifications to the manuscript to eliminate any misunderstandings regarding the specifics of the presented models.**

Below, we provide a detailed description of the subsystem blocks used in the model (Figure 4), as well as the demodulator subsystem presented in Figure 5.

Initialization of block parameters is performed by clicking on the block icon in the Simulink environment. This interactive approach enables on-the-fly configuration of parameters during experimentation. Additionally, clicking on the arrow icon within a Simulink block structure provides access to the internal architecture of the respective subsystem. In the following, we elaborate on the functionality and structure of the main blocks utilized in the proposed models.

The Inport block imports the external RF signal into the Simulink environment, serving as the input source for subsequent processing stages. The LNA amplifies the signal to a level suitable for further stages while minimizing added noise. The RF Demodulator performs frequency downconversion, shifting the RF signal to an intermediate frequency (IF), thus preparing it for digital signal processing and data extraction. We highlight that the demodulator subsystem (Figure 5) includes a LO that allows the simulation of phase noise, contributing to quadrature instability, as well as a block for applying phase shifts to the I/Q channels.

The VGA dynamically adjusts the gain to optimize signal amplitude for downstream processing (e.g., analog-to-digital conversion or decoding). After initial amplification by the LNA, the signal level may fluctuate due to varying reception conditions or circuit losses. VGA ensures amplitude remains within the optimal dynamic range, preserving signal quality. The Outport block outputs the processed signal—either in the form of demodulated data or intermediate results—for further analysis or integration into other subsystems.

Accurate demodulation in 5G receivers requires precise separation of the incoming RF signal into in-phase (I) and quadrature (Q) components. The proposed model enables programmable simulation of impairments affecting quadrature components, specifically evaluating the impact of I/Q gain mismatch, which causes signal constellation distortion, complicating symbol detection and degrading rejection of image frequencies. We also assessed the effects of I/Q phase mismatch, which results in rotation and displacement of the signal constellation, impairing accurate information recovery. For an RF demodulator, maintaining strict orthogonality between I and Q is critical. Any mismatch may lead to phase crossing and significantly reduce demodulation accuracy.

To mitigate the effects of both, gain and phase mismatches, we employed a combination of digital compensation techniques and calibration algorithms. Adaptive correction algorithms were implemented to estimate and compensate for these impairments based on real-time signal analysis. Specifically, we explored blind IQ correction techniques that rely on statistical properties of the signal to estimate deviations and apply corrective adjustments in the digital domain. Additionally, we investigated the use of pilot signals—known patterns introduced into the transmission—which help estimate I/Q channel parameters with higher precision and enable improved calibration. Finally, from a hardware implementation perspective, we consider the integration of compensation modules into FPGA or DSP platforms to be highly promising. Such integration enables real-time correction through a combination of hardware-assisted preprocessing and digital fine-tuning, making the receiver more robust under dynamic channel conditions.
